# Supplementary material for: Considering Two Aspects of Fish Welfare on African Catfish (Clarias gariepinus) Fillet throughout Postmortem Condition: Efficiency and Mechanisms
Source: Foods. 2022 Dec 17;11(24):4090. doi: 10.3390/foods11244090 (PMC9778388; doi:10.3390/foods11244090)
Supplement: Supplementary file 1 [file foods-11-04090-s001.zip › foods-1974084-supplementary.pdf]

Supplementary for (Hematyar, Consequences of fish welfare on African catfish (*Clarias gariepinus*) fillet throughout postmortem condition: efficiency and mechanisms)

Table S1. The number of fillets for each group.

	T0	T3	T6	T9
**D90**	B=6<br>Unb=6	B=6<br>Unb=6	B=6<br>Unb=6	B=6<br>Unb=6
**D120**	B=6<br>Unb=6	B=6<br>Unb=6	B=6<br>Unb=6	B=6<br>Unb=6
**D150**	B=6<br>Unb=6	B=6<br>Unb=6	B=6<br>Unb=6	B=6<br>Unb=6

B: Bled, Unb: Un-bled, D: Density, T: Time

Table S2: Three-Way ANOVA p-value table for the data variables

Effect of (p-value)→ / Variable ↓		Stocking density	Slaughtering method	Storage time	Stocking density x Slaughtering method	Stocking density x Storage time	Slaughtering method x Storage time	Stocking density x Slaughtering method x Storage time
**Rigor index**		0.001	<0.0001	<0.0001	0.055	<0.0001	<0.0001	<0.0001
**pH**		0.166	<0.001	<0.0001	0.555	<0.0001	<0.0001	<0.0001
**Calpain activity**		<0.05	0.3590	<0.0001	0.7160	<0.001	0.8550	0.6870
**Hardness analysis**		<0.0001	<0.001	<0.0001	0.4110	<0.01	<0.01	<0.05
**Liquid loss**		0.142	<0.001	<0.0001	0.414	0.391	0.092	0.224
**Haem content**		<0.001	<0.0001	<0.0001	<0.001	0.20	<0.0001	0.232
**Lipid and protein oxidation**	**TBARS**	0.313	<0.001	<0.0001	0.219	0.252	<0.001	0.703
	**Carbonyl content**	0.793	<0.0001	<0.0001	<0.05	0.268	<0.0001	0.418

Significance interactions <0.05, <0.01, <0.001, <0.0001 are indicated as the green colour cells.

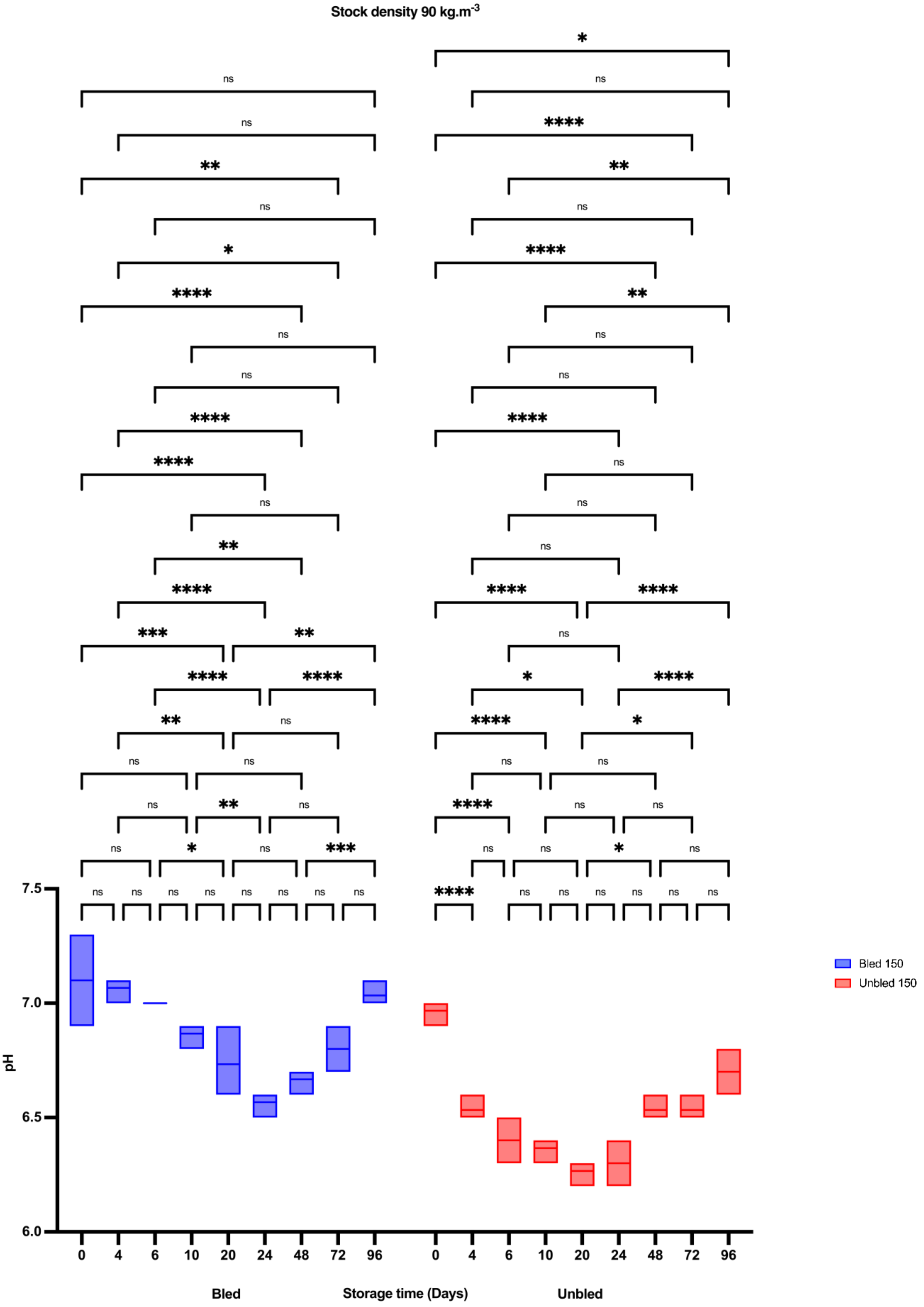

Figure S1A. pH: During the storage time (0, 4, 6, 10, 20, 24, 46, 72, and 96 Days) in bled and un-bled groups at different stock density 90 kg.m^{-3}; ($p < 0.05$, Tukey's multiple comparisons test; ns: non-significant; * $p < 0.05$; ** $p < 0.01$; *** $p < 0.001$; ****$p < 0.0001$). Box plots showing line at mean.

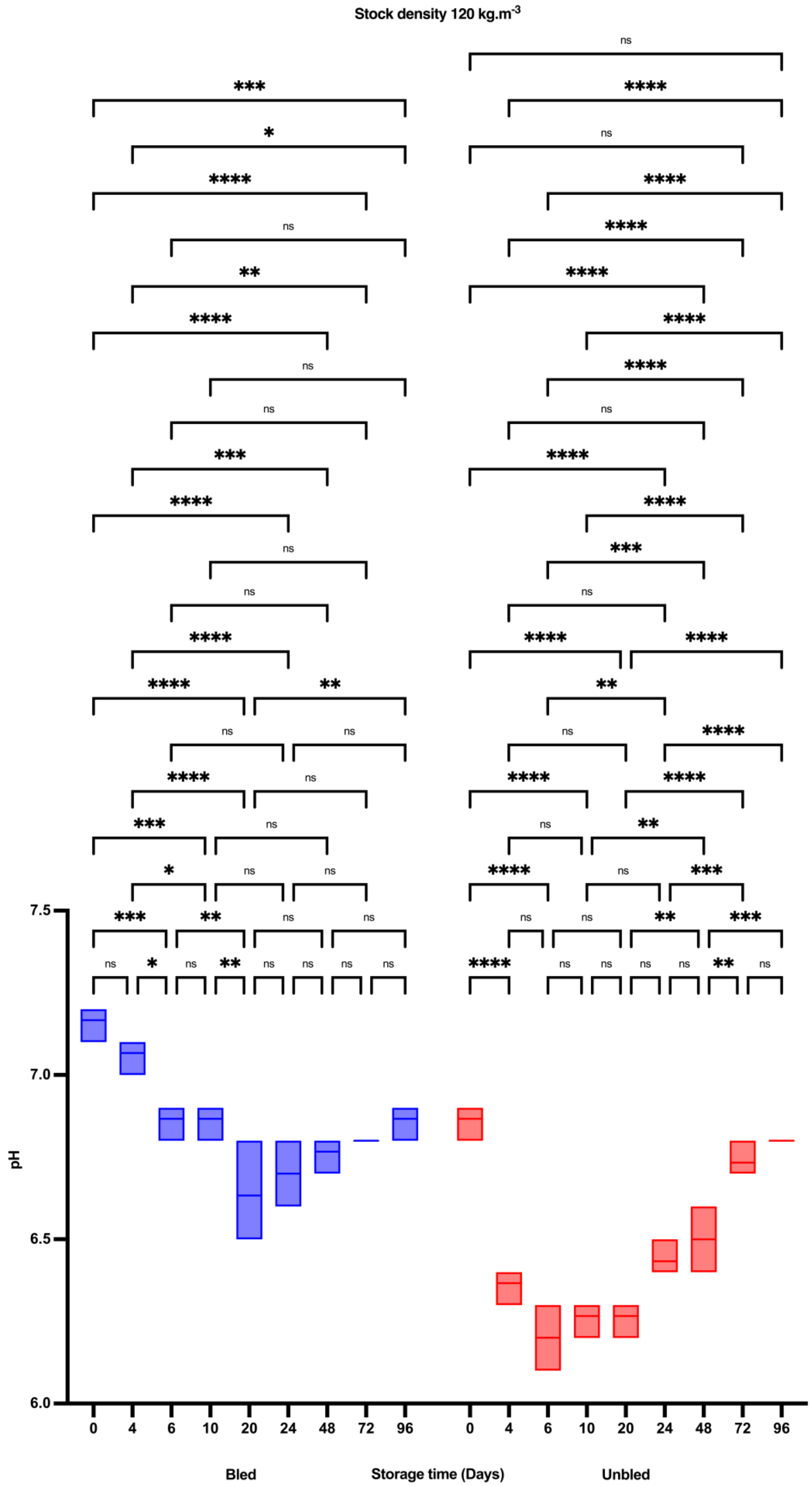

Figure S1B. pH: During the storage time (0, 4, 6, 10, 20, 24, 46, 72, and 96 Days) in bled and un-bled groups at different stock density 120 kg.m^{-3}; ($p < 0.05$, Tukey's multiple comparisons test; ns: non-significant; * $p < 0.05$; ** $p < 0.01$; *** $p < 0.001$; ****$p < 0.0001$). Box plots showing line at mean.

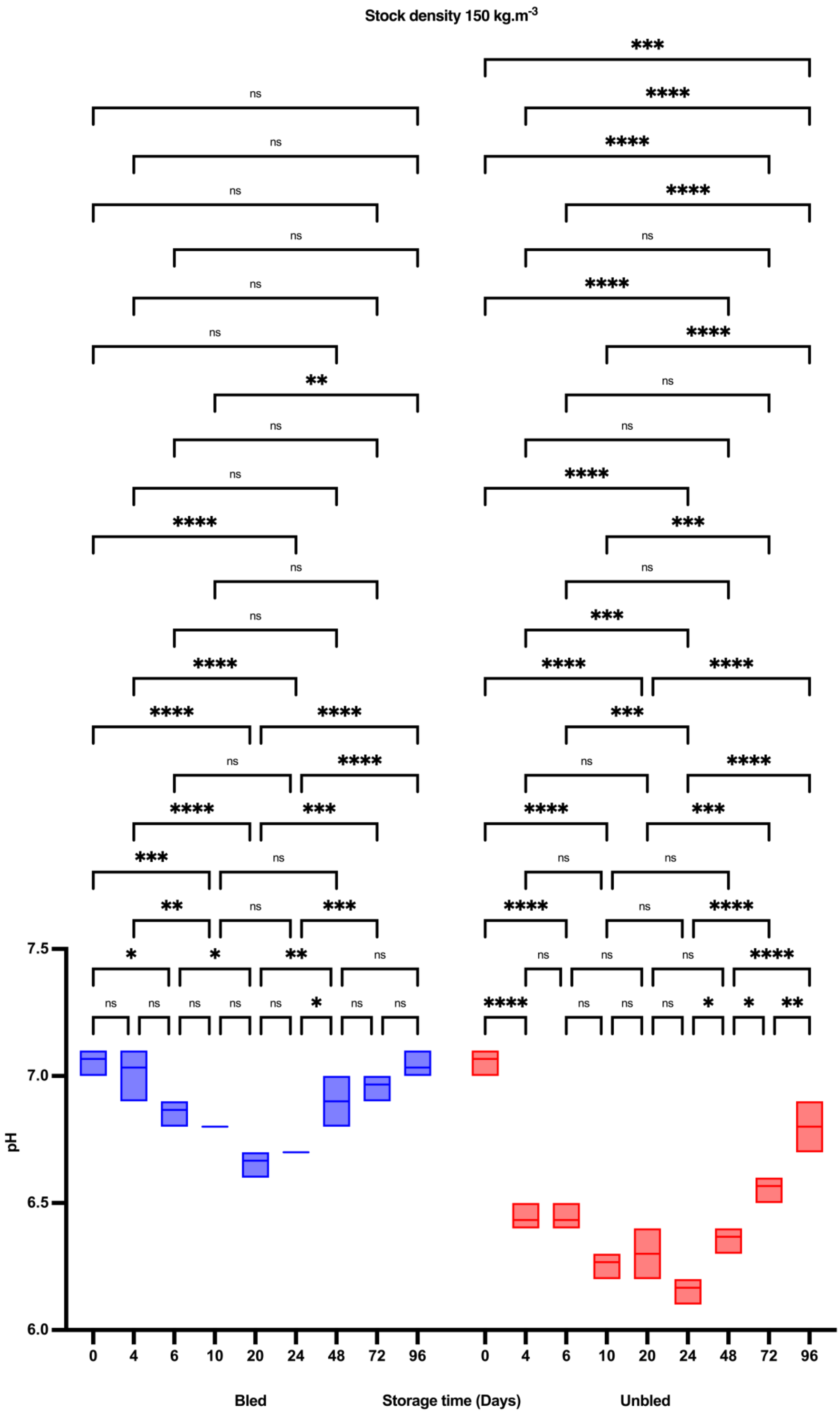

Figure S1C. pH: During the storage time (0, 4, 6, 10, 20, 24, 46, 72, and 96 Days) in bled and un-bled groups at different stock density 150 kg.m^{-3}; ($p < 0.05$, Tukey's multiple comparisons test; ns: non-significant; * $p < 0.05$; ** $p < 0.01$; *** $p < 0.001$; ****$p < 0.0001$). Box plots showing line at mean.

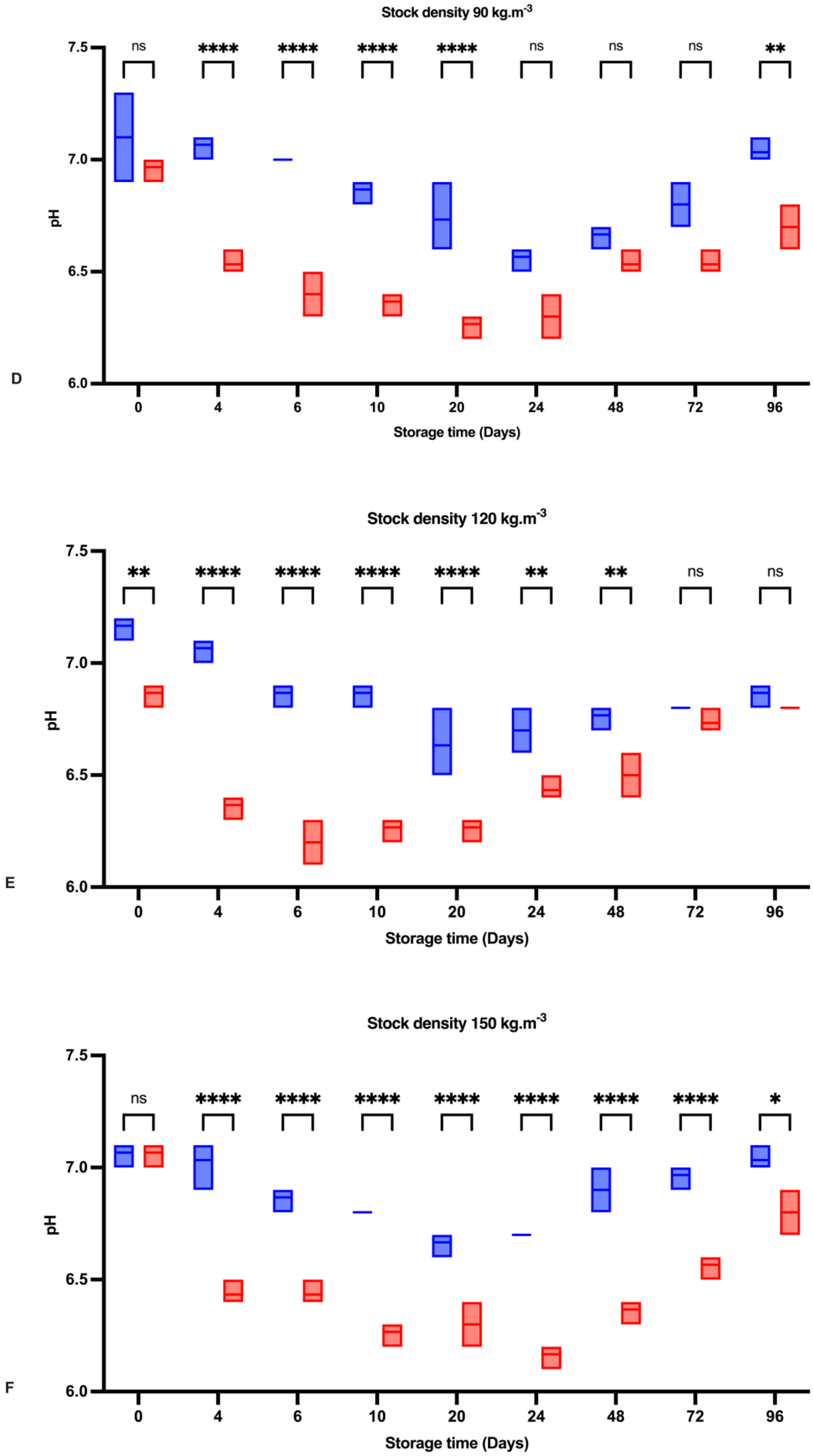

Figure S1DEF. pH: In bled and un-bled groups at each storage time (0, 4, 6, 10, 20, 24, 46, 72, and 96 Days) and at different stock densities (90, 120 and 150 kg.m^{-3}); ($p < 0.05$, Tukey's multiple comparisons test; ns: non-significant; * $p < 0.05$; ** $p < 0.01$; *** $p < 0.001$; ****$p < 0.0001$). Box plots showing line at mean.

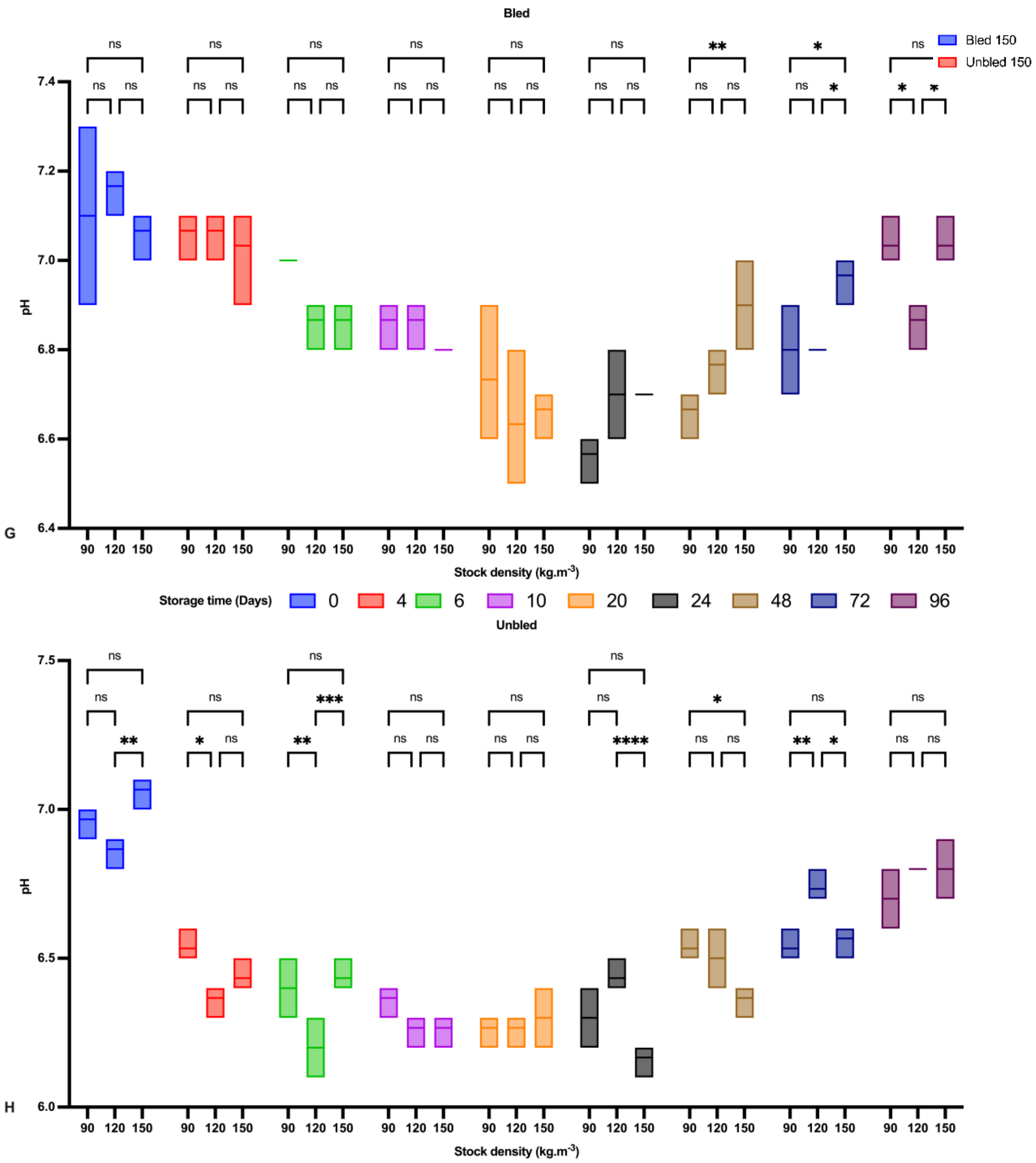

Figure S1GH. pH: In different stocking densities (90, 120 and 150 kg.m^{-3}) during storage time (0, 4, 6, 10, 20, 24, 46, 72, and 96 Days) in bled and un-bled groups; ($p < 0.05$, Tukey's multiple comparisons test; ns: non-significant; * $p < 0.05$; ** $p < 0.01$; *** $p < 0.001$; ****$p < 0.0001$). Box plots showing line at mean.

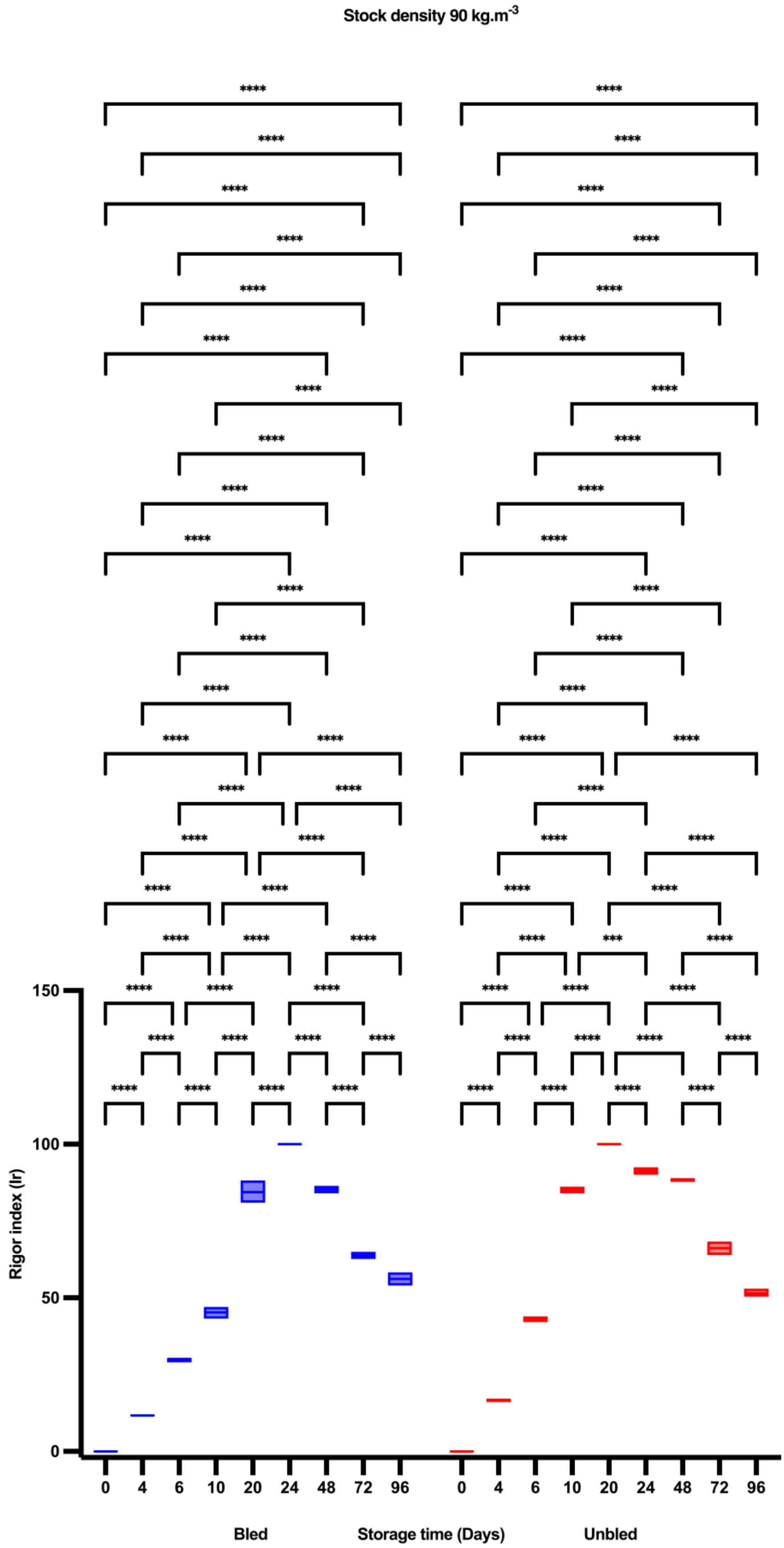

Figure S2A. Rigor index: During the storage time (0, 4, 6, 10, 20, 24, 46, 72, and 96 Days) in bled and un-bled groups at different stock density 90 kg.m^{-3}; ($p < 0.05$, Tukey's multiple comparisons test; ns: non-significant; * $p < 0.05$; ** $p < 0.01$; *** $p < 0.001$; ****$p < 0.0001$). Box plots showing line at mean.

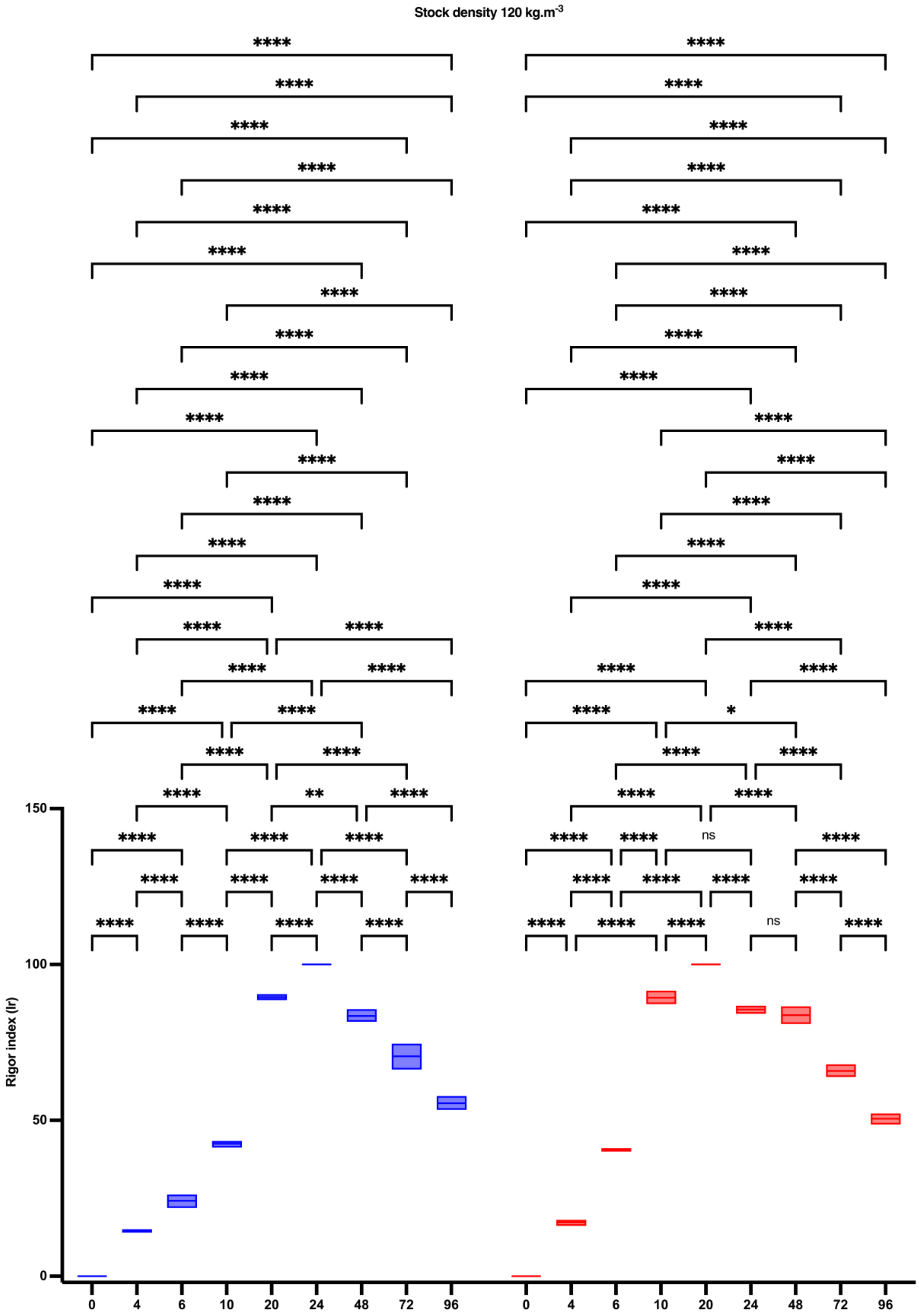

Figure S2B. Rigor index: During the storage time (0, 4, 6, 10, 20, 24, 46, 72, and 96 Days) in bled and un-bled groups at different stock density 120 kg.m^{-3}; ($p < 0.05$, Tukey's multiple comparisons test; ns: non-significant; * $p < 0.05$; ** $p < 0.01$; *** $p < 0.001$; ****$p < 0.0001$). Box plots showing line at mean.

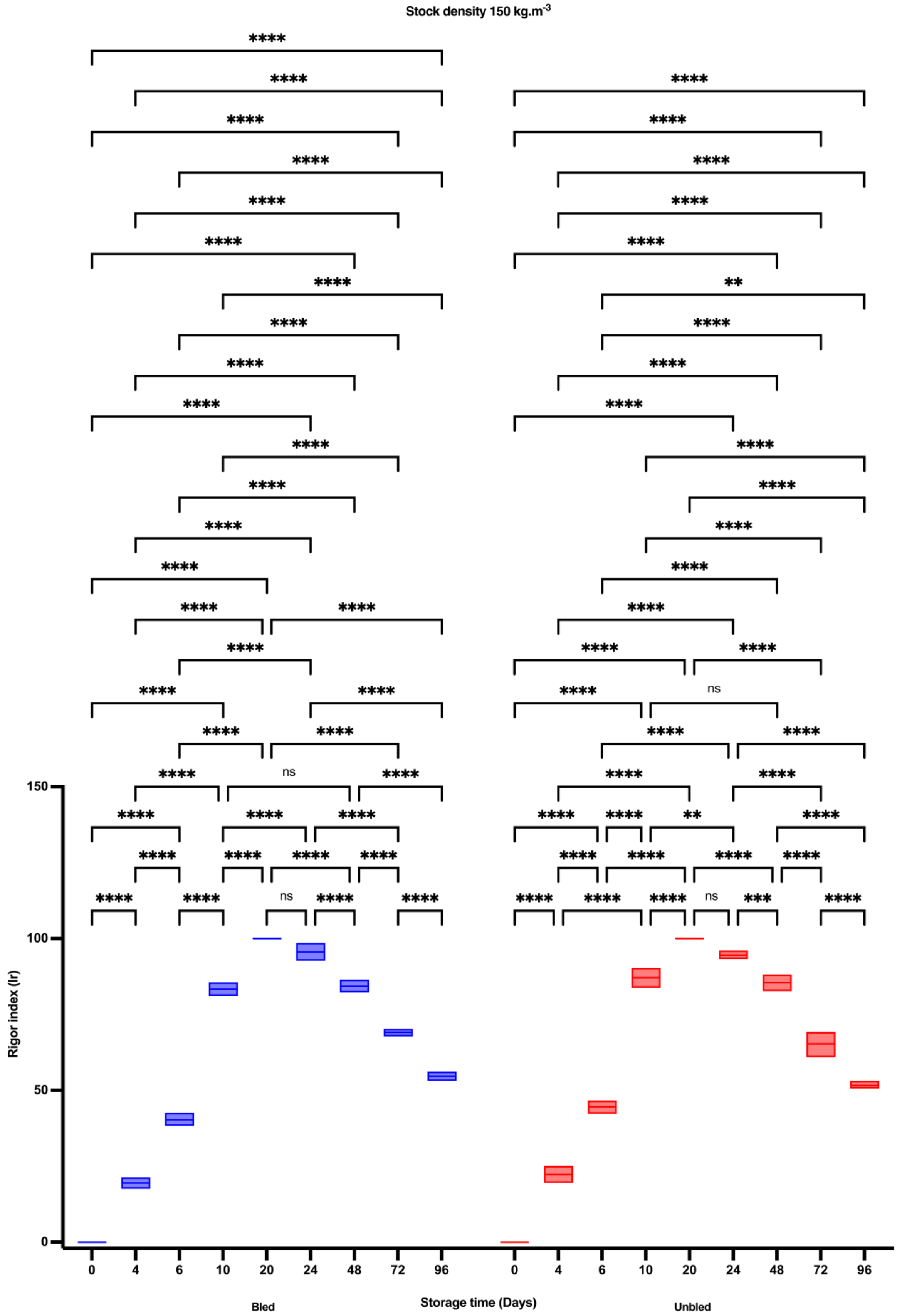

Figure S2C. Rigor index: During the storage time (0, 4, 6, 10, 20, 24, 46, 72, and 96 Days) in bled and un-bled groups at different stock density 150 kg.m^{-3}; ($p < 0.05$, Tukey's multiple comparisons test; ns: non-significant; * $p < 0.05$; ** $p < 0.01$; *** $p < 0.001$; ****$p < 0.0001$). Box plots showing line at mean.

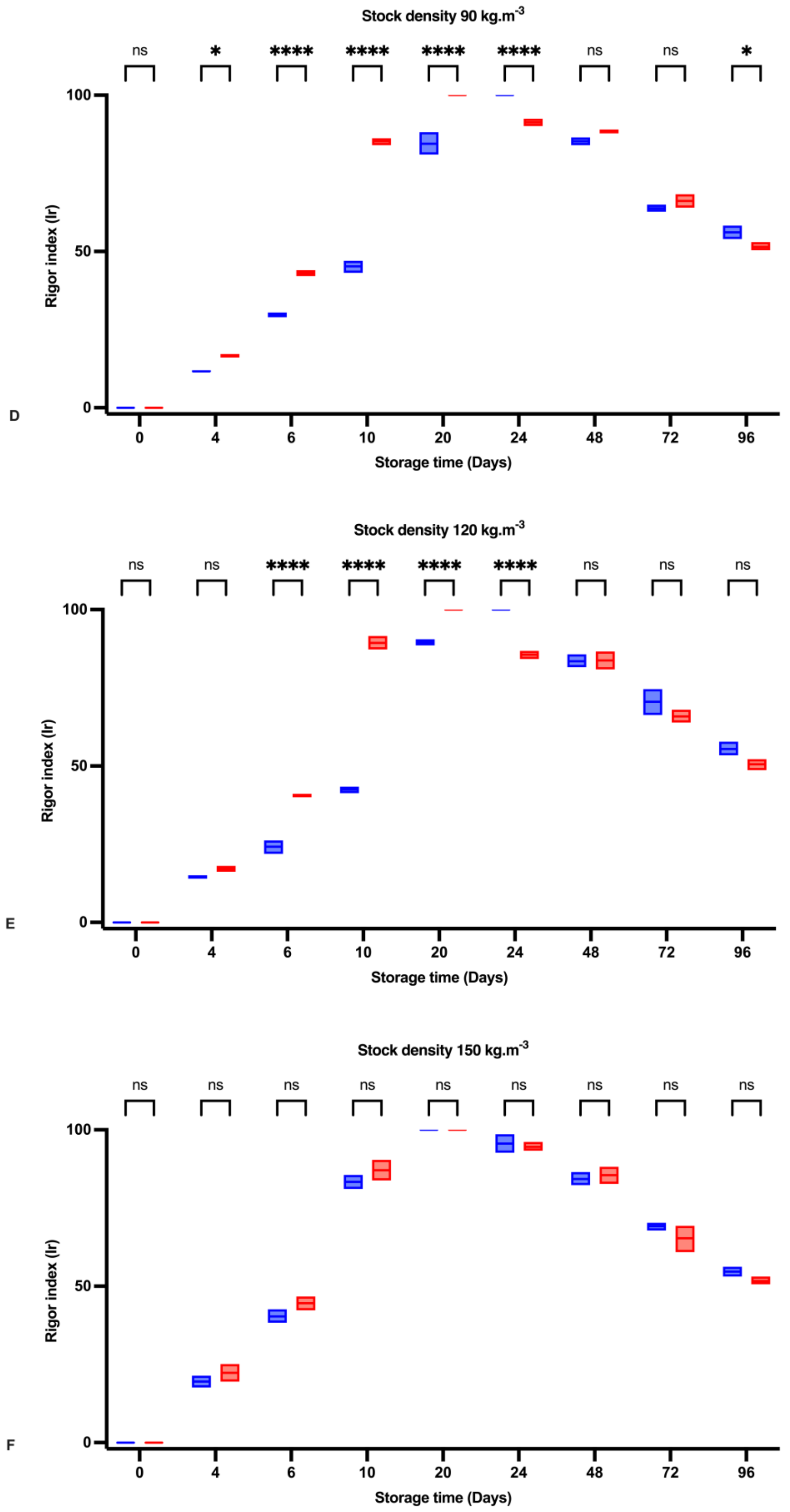

Figure S2DEF. Rigor index: In bled and un-bled groups at each storage time (0, 4, 6, 10, 20, 24, 46, 72, and 96 Days) and at different stock densities (90, 120 and 150 kg.m^{-3}); ($p < 0.05$, Tukey's multiple comparisons test; ns: non-significant; * $p < 0.05$; ** $p < 0.01$; *** $p < 0.001$; ****$p < 0.0001$). Box plots showing line at mean.

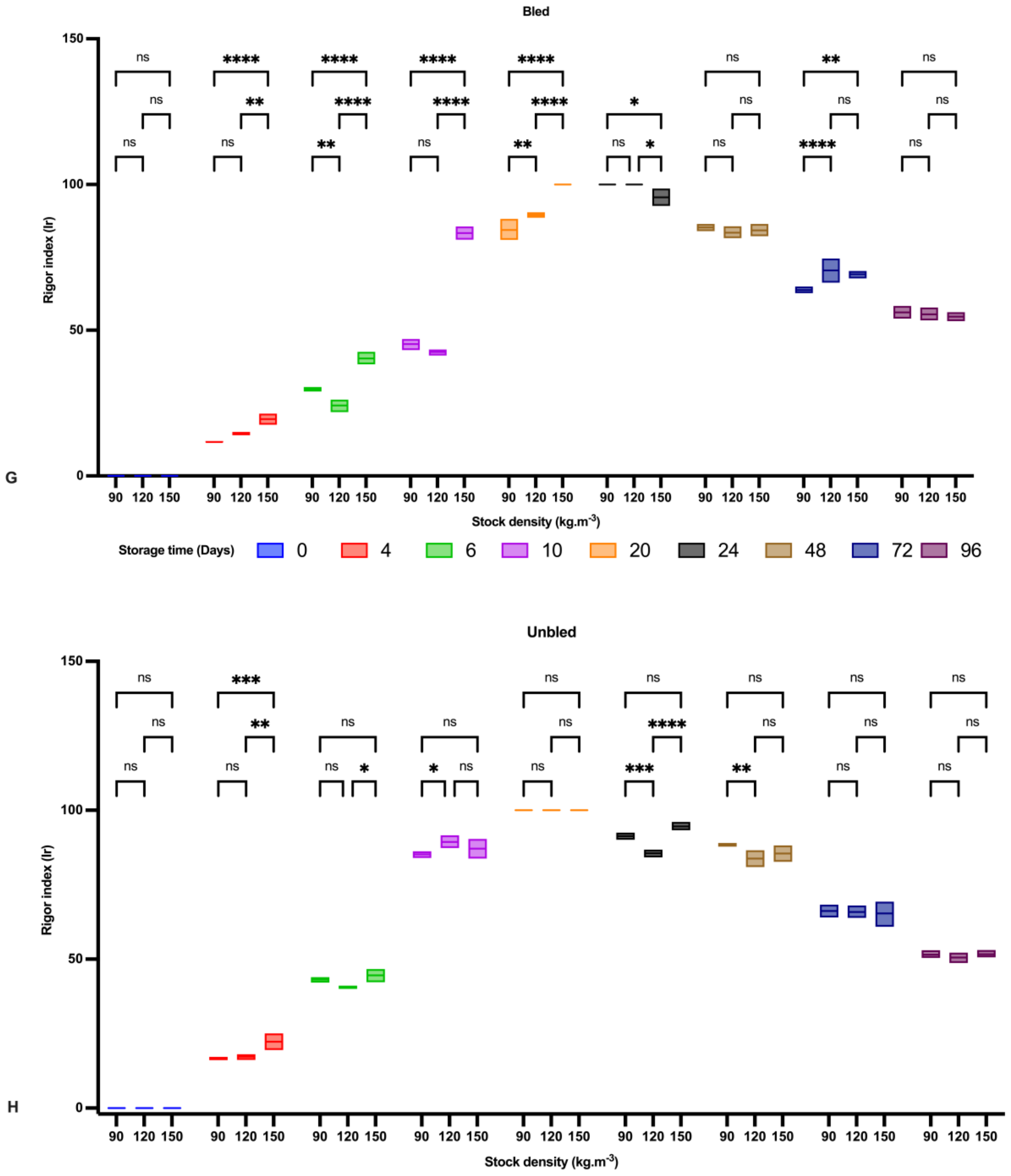

Figure S2GH. Rigor index: In different stocking densities (90, 120 and 150 kg.m^{-3}) during storage time (0, 4, 6, 10, 20, 24, 46, 72, and 96 Days) in bled and un-bled groups; ($p < 0.05$, Tukey's multiple comparisons test; ns: non-significant; * $p < 0.05$; ** $p < 0.01$; *** $p < 0.001$; ****$p < 0.0001$). Box plots showing line at mean.
